# Orellanine: From Fungal Origin to a Potential Future Cancer Treatment

**DOI:** 10.1021/acs.jnatprod.2c01068

**Published:** 2023-06-12

**Authors:** Mark J. Lyons, Carsten Ehrhardt, John J. Walsh

**Affiliations:** School of Pharmacy and Pharmaceutical Sciences, Trinity College Dublin, Dublin 2, Ireland

## Abstract

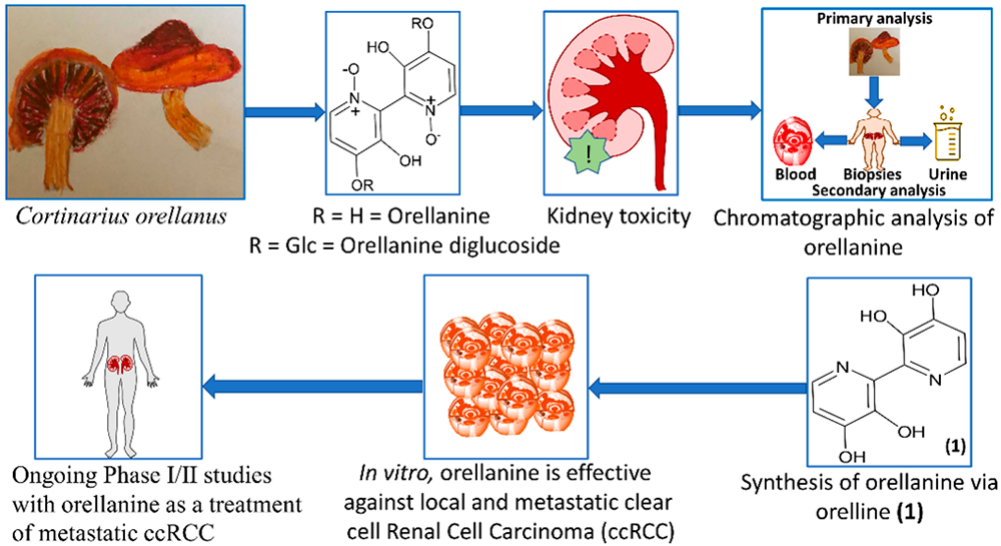

Fungal metabolites represent an underutilized resource in the development of novel anticancer drugs. This review will focus on the promising fungal nephrotoxin orellanine, found in mushrooms including *Cortinarius orellanus* (Fools webcap). Emphasis will be placed on its historical significance, structural features, and associated toxicomechanics. Chromatographic methods for analysis of the compound and its metabolites, its synthesis, and chemotherapeutic potential are also discussed. Although orellanine’s exceptional selectivity for proximal tubular cells is well documented, the mechanics of its toxicity in kidney tissue remains disputed. Here, the most commonly proposed hypotheses are detailed in the context of the molecule’s structure, the symptoms seen following ingestion, and its characteristic prolonged latency period. Chromatographic analysis of orellanine and its related substances remains challenging, while biological evaluation of the compound is complicated by uncertainty regarding the role of active metabolites. This has limited efforts to structurally refine the molecule; despite numerous established methods for its synthesis, there is minimal published material on how orellanine’s structure might be optimized for therapeutic use. Despite these obstacles, orellanine has generated promising data in preclinical studies of metastatic clear cell renal cell carcinoma, leading to the early 2022 announcement of phase I/II trials in humans.

One of the key discoveries made following the examination of the Neanderthal remains found in the El Sidrón cave, Spain, was the presence of plant- and mold-based medicinal compounds in the plaque of one of the individuals that was unearthed.^1^ This provides perhaps the earliest evidence to date of the service that nature has given us as a provider of medicinal products. It may be tempting to propose that such naturally inspired molecules, many of which are small molecular agents (SMAs), have diminishing relevance to modern medicine, with the rise of rational drug design, biologicals, and most recently, cell- and gene-based therapies, all providing new avenues through which to approach the treatment/management of disease and the maintenance of health. However, given that sources of both salicylic acid and penicillins were present in Neanderthal plaque and the importance that both aspirin and penicillins still hold in modern medicine, there may still be a significant role for naturally inspired SMAs to play. Indeed, the growing popularity of antibody drug conjugates (ADCs),^2^ incorporating both a biological and a (typically naturally inspired) SMA suggests that there is potential for such molecules to be deployed in new and innovative ways. ADCs typically incorporate a potent cytotoxin that lacks the selectivity to be used in patients, coupled to an antibody targeting an antigen expressed on the cell type of interest. These two components are joined by a linker unit, which in most cases is designed to preferentially cleave inside the tumor cell, thus limiting off target toxicity.^3^ This approach has enabled the use of highly potent SMAs such as the auristatin, maytansinoid, pyrrolebenozodiazepine, and calicheamicin series of warheads. As of 2023, there have been 15 different ADCs that have held approvals for the treatment of a range of both solid and nonsolid tumors, with 10 such products being approved between 2019 and 2023.^4,5^ The synergy between naturally inspired SMAs and ADC systems is further reinforced by the fact that such compounds have been utilized as warheads in 14 of the 15 approved ADCs.

As highlighted by the ADCs, one field where naturally inspired SMAs remain especially relevant is chemotherapeutics. From 1981 to 2019, 285 new chemical entities were approved with an authorization for use as antitumor agents. Of these, just over 30% were either natural products or were structurally derived from such compounds.^6^ This encompasses agents such as the taxanes, dolastatins, and calicheamicins, with sources from the plant (*Taxus brevifolia*), animal (*Dolabella auricularia*), and bacterial (*Micromonospora echinaspora*) kingdoms, respectively. However, despite its significant contributions to other fields of medicine (most notably antimicrobials), when it comes to natural sources of clinically approved anticancer agents, the kingdom of fungi is conspicuous by its absence. While fungal endophytes have played a notable role in the discovery of a number of antitumor agents, this has traditionally been via symbiotic relationships with a larger organism, to which the compound is then attributed (e.g., paclitaxel and *Taxus brevifolia*);^7^ there has yet to be an approved anticancer agent that is extracted/derived from an independent fungal source.^7−10^ This is not to relay that there is a lack of candidate molecules and therapies—as of 2015, Kornienko et al.^10^ reported 19 different in-human studies conducted to assess the efficacy of fungal metabolites in the treatment of cancer. Promising agents currently in clinical trials include plinabulin, a semisynthetic analogue of the seaweed-derived fungal metabolite halimide, which is found in a marine strain of *Aspergillus* sp.^11^ By binding to the colchicine binding site on β-tubulin, it disrupts microtubule stability in angiogenic vasculature and has shown a promising toxicity to efficacy profile when used with docetaxel in phase I clinical trials for patients with nonsmall cell lung cancer (NSCLC).^12^ Another agent that has shown promise in early phase clinical trials is PX-866, a synthetic derivative of the furanosteroid wortmannin.^13^ This fungal metabolite, produced by a strain *of Penicillium wortmannii*, is an irreversible PI3K inhibitor, which has shown efficacy in a range of solid tumors and is currently undergoing phase II trials to investigate its efficacy in the treatment of glioblastoma^14^ and castration-resistant prostate cancer.^15^ There are also a growing number of cases where modern technologies and targeting approaches are being employed to refine the cytotoxicity of fungal metabolites that had previously been deemed unsuitable for clinical use. One of the classes of agents currently being investigated are antibody-targeted amanitin conjugates (ATACs). The amatoxins (Figure 1) are widely recognized as the most lethal of the mushroom toxins, acting as RNA polymerase II inhibitors.^16^

**Figure 1:**
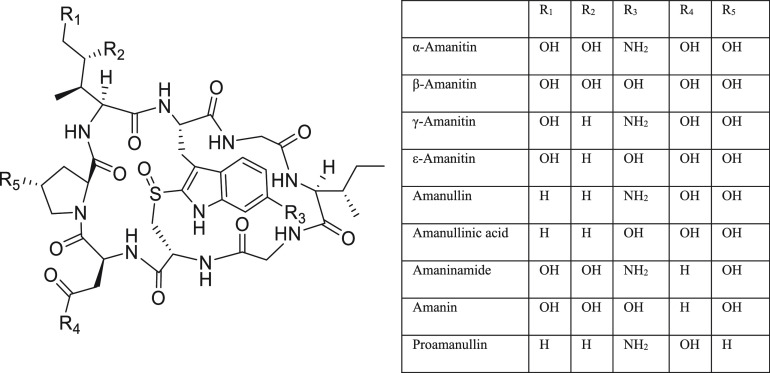
Amatoxin structures.

As the most potent of the amatoxins, α-amanitin and β-amanitin are found in a number of the most toxic known mushrooms, including *Conocybe filaris* and all species in the *Galerina* genus.^17^ In an attempt to improve the selectivity of these molecules as potential antitumor agents, there has been a number of recent studies focused on the development of ATACs.^18−22^ These are ADCs, which incorporate analogues of the fungal-derived bicyclic octapeptides, α-amanitin and/or β-amanitin, as the cytotoxic warhead. Conjugation to an antibody not only allows for targeted delivery to specific cell types but also stops the toxin from acting as an OATP1B3 substrate, greatly reducing hepatotoxicity. Another fungal metabolite that had previously shown use limiting toxicity was irvofulven, an analogue of the sesquiterpene illudin S, found primarily in *Omphalotus illudens*. A clinical study in patients with recurrent ovarian cancer reported dose-limiting retinal toxicity, failing to progress beyond phase II trials.^23^ Recent advances in screening technology have since revealed that irvofulven is particularly effective in tumors with a nucleoside excision repair (NER) deficiency, which has led to a revival of interest in the compound.^24^ Even within this limited selection of examples, the range of different structural scaffolds and novel mechanisms of action afforded by fungal metabolites is apparent. The latter two examples also highlight the potential for synergy that exists between fungal-derived SMAs and modern drug development technologies. This review will focus on orellanine, a toxic mushroom metabolite that has shown promise as a potential treatment for clear cell renal cell carcinoma (ccRCC), based on preclinical studies. Its historical significance, structural features, and mechanism of action (MOA) will be discussed, with further attention given to the potential activity of its metabolites and its selectivity for proximal tubular cells. A critical analysis of published methods for its analysis will be covered, together with a discussion on approaches used for orellanine synthesis. Exploration of these topics will form the basis for highlighting the significant challenges that must still be addressed to understand and optimize orellanine’s potential as an anticancer agent.

## Identity and Toxicity

### Discovery as a Toxin

The toxicity of mushrooms from the *Cortinarius* genus first came to public attention in 1957, following the mass poisoning of 135 people in Poland.^25^ This incident was attributed to *Cortinarius orellanus* (fool’s webcap); while the toxin has yet to be identified outside of the *Cortinarius* genus, it has been found in other members of the *Cortinarius* family, most notably *Cortinarius rubellus* (deadly webcap), which has since been linked to several poisonings throughout Europe and North America.^26^ Although confusion with members of the *Psilocybe* genus has been reported,^27−30^ the mushrooms are most commonly ingested in the belief that they are a different species of edible mushroom, such as *Craterellus tubaeformis* (yellow foot)^31^ or *Cantharellus cibarius* (golden chanterelle).^32^

**Figure 2:**
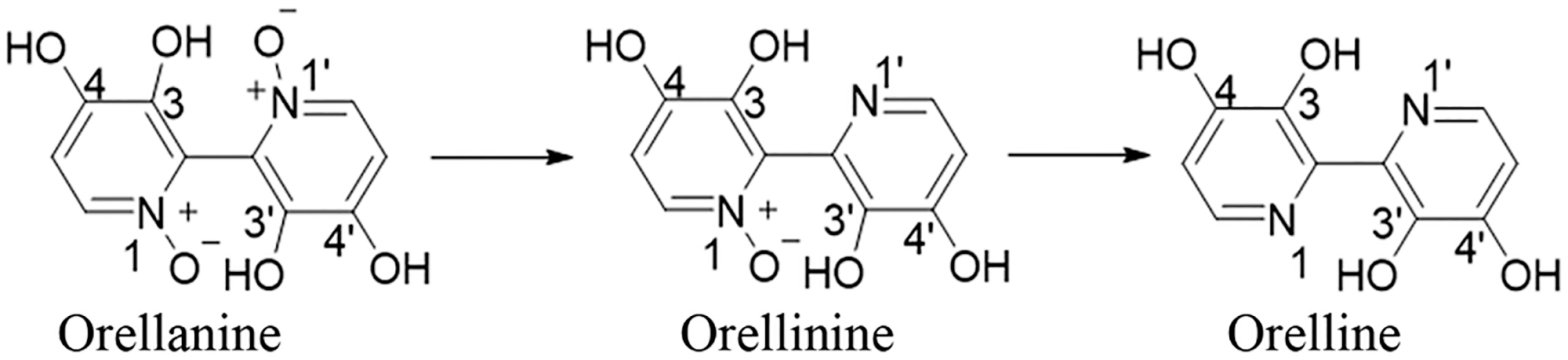
Structures left to right of orellanine, orellinine, and orelline.

In response to the dangers posed by such inadvertent ingestion, studies have been performed to better understand the nature of the toxin involved. Some earlier work attributed the toxicity of these mushrooms to a series of fluorescent cyclopeptides dubbed the cortinarins,^33^ but these results have never been successfully replicated and the existence of such compounds remains in doubt.^34,35^ While such a peptide-based structure aligns with other fungal poisons such as the amatoxins (Figure 1), the toxin present in certain members of the *Cortinarius* genus is structurally quite different. It was only in 1962 that the actual toxicant, orellanine (3,3′-4,4′-tetrahydroxy-(2,2′-bipyridine)-1,1′-*N*-oxide), was first isolated from *C*. *orellanus*.^36^ It was not until 1979 that its identity was confirmed^37^ (Figure 2).

### Structural Features and Associated Toxicomechanics

**Figure 3:**
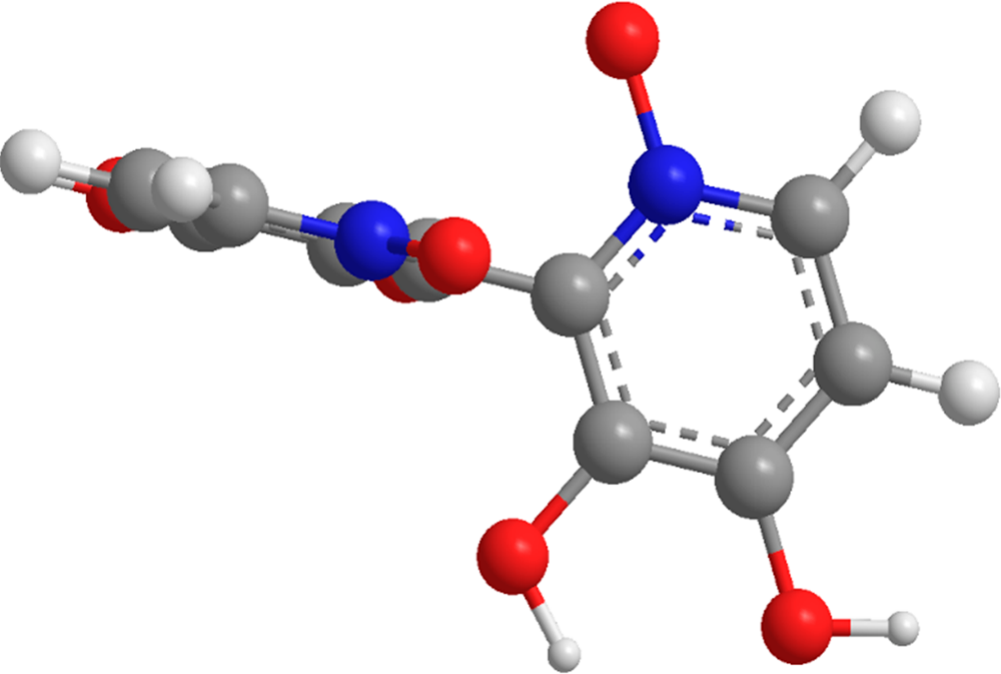
Illustration of the dihedral angle in orellanine.

A notable feature of its structure is the presence of a tetrahydroxy bipyridine *N*-oxide scaffold. The two *N*-oxide groups have a significant impact on the conformation of orellanine. While bipyridines in general tend to adopt synperiplanar conformations,^38^ orellanine maintains an antiperiplanar configuration as this both allows for hydrogen bonding between *N*-oxide and the neighboring 3/3′ OH and avoids an unfavorable dipole–dipole clash between the *N*-oxides.^39^ X-ray crystallography suggests that orellanine adopts a conformation where the planes of the two pyridine *N*-oxide rings are almost perpendicular to each other^40,41^ (Figure 3).

The stability profile of orellanine is also unusual; while *N*-oxides typically have excellent thermostability, orellanine decomposes when heated above 150 °C.^37,40,42^ Rapid reduction also occurs following exposure to UV radiation.^37,42,43^ This reaction mechanism has been studied in detail and is believed to be facilitated by interaction between the *N*-oxides and the 3,3′-hydroxy groups, allowing for stepwise reduction of the *N*-oxides.^39^ This results first in the formation of the active breakdown product orellinine, which itself is then rapidly converted to the inactive orelline^39,40,42−46^ (Figure 2). This suggests an essential role for the *N*-oxide functionality in the mechanism of orellanine toxicity in renal cells, although the exact mechanism is not fully understood. Any future refinement of the selectivity and/or optimization of the *in vivo* activity of orellanine will require a greater understanding of the role of the *N*-oxides in its MOA.

**Figure 4:**
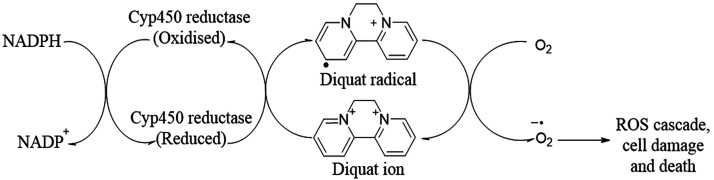
Redox cycling of diquat.

**Figure 5:**
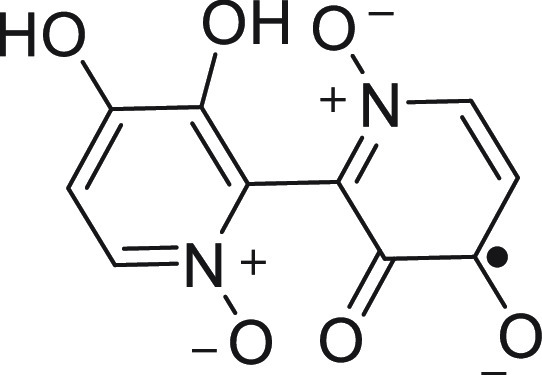
*ortho*-Semiquinone radical of orellanine.

Due to structural similarities, orellanine has been compared to the herbicides diquat and paraquat, with some proposing a shared MOA^47,48^ (Figure 4),

This would entail reduction followed by formation of a stable free radical, allowing for redox cycling, resulting in the formation of reactive oxygen species (ROS) and depletion of cellular NADPH.^40,49,50^ Orellanine’s toxicity in photosynthesizing cells and organisms^48,51^ would seem to suggest a similarly nontargeted MOA. However, the fungal toxin was demonstrated to lack paraquat’s capacity to interfere directly with the electron transport chain of chloroplasts,^51^ and early cyclic voltammetry studies showed significantly different redox profiles for orellanine and the bipyridine herbicides.^52,53^ Indeed, although orellanine’s *N*-oxides do undergo reduction, cyclic voltammetry studies indicate a lack of reoxidation seen in the action of the bipyridine herbicides.^53^ Instead, orellanine has been shown to undergo both one- and two-electron oxidations via a number of different pathways.^54^ One of the most obvious structural explanations for any differences in toxicomechanics would be the presence of additional functional groups at the meta and para positions. In the case of orellanine, the presence of the catechol groups on the pyridine *N*-oxide rings at these positions has been shown to allow for chelation of metal ions, particularly iron, resulting in the generation of ROS^54−57^ and DNA scission.^56^ They also facilitate the formation of an *ortho*-semiquinone radical (Figure 5), which has been suggested to facilitate both redox cycling and covalent adduction of essential cellular structures,^40,45,58^ leading to a more multifaceted action profile than the bipyridyl herbicides.

The complexity of orellanine’s toxicity is highlighted by the fact that despite the extensive oxidative damage caused in rat models, key components of the cell’s antioxidant defenses were found to be downregulated following treatment with orellanine.^59^ Although this phenomenon is not yet fully understood, it suggests a capacity for orellanine to selectively suppress the expression of certain enzymes, further increasing the cell’s susceptibility to the oxidative damage. Earlier studies have also shown an ability of orellanine to affect the mitochondrial electron transport chain, acting as a noncompetitive inhibitor of alkaline phosphatase, at levels not seen in the toxicity of classic bipyridine toxins.^60,61^

In contrast to the uncertainty surrounding the exact mechanism of orellanine’s toxicity, its site of action is understood to a far greater degree. A combination of animal studies and biopsies taken from poisoned patients have shown that orellanine acts on the proximal tubular cells of the kidney, with a significant degree of selectivity, even over other renal cells.^59,62^ The selectivity of its uptake is poorly understood—while it has been suggested that it enters proximal tubular cells via an active, sodium independent transporter,^63^ this has yet to be confirmed or investigated further. It has also been suggested that orellanine is re-absorbed from the urine by a specific renal transporter, as the observed plasma half-life in rats was longer than what would be predicted for a molecule with similar physicochemical properties.^43^

Indeed, there is still debate regarding the rate at which orellanine is cleared from bodily fluids. Some studies detected orellanine in plasma, whole blood, and urine several days post-ingestion,^64,65^ while others state that it was undetectable in both blood and urine 2–3 days post-ingestion.^66,67^ There is also uncertainty regarding the retention of orellanine within the kidneys themselves. It was believed to be strongly retained within proximal tubular cells, with significant levels reported in human biopsies taken over 6 months after initial fungal ingestion.^64^ This observation has not been supported by the work of Flament et al., who reported an absence of orellanine across five renal biopsies taken between 6- and 55-days postingestion.^68^ While the duration of orellanine retention within the kidneys is unclear, its initial uptake appears to be rapid in nature. A study by Nieminen et al.^69^ found that co-administration of furosemide in rats led to increased orellanine toxicity in the proximal tubules. As furosemide is completely cleared from rats within 5 h, these results suggest that orellanine begins to accumulate in renal tissues within hours of ingestion. This is further supported by a case study which reported that even when plasmapheresis was initiated within 44 h of *C*. *orellanus* ingestion, the patient still experienced significant renal buildup of orellanine and subsequent kidney failure.^70^ While toxicology studies suggest that high micromolar levels are required to precipitate kidney failure in humans,^31^ the extended retention of orellanine may facilitate a cumulative MOA where toxic effects build up over time. This could explain the slow onset of symptoms following orellanine poisoning. Initial symptoms tend to be gastric in nature, normally occurring on day 3 post-ingestion and typically manifesting as nausea, vomiting, and diarrhea, although abdominal cramps and anorexia have also been reported.^71^ Given the corrosive effects that the bipyridyl herbicides exert on GI mucosal surfaces,^49,50^ these symptoms could be seen as indicative of some form of GI toxicity. However, a study using a murine model found that following subcutaneous administration of orellanine, there was no epithelial necrosis seen along the GI tract, with the only visible damage being the presence of gastric lesions in some, but not all of the mice studied.^72^ In addition to this is the evidence provided by a follow-up study in patients who had experienced orellanine poisoning.^73^ When compared to patients suffering from chronic kidney disease or requiring dialysis for reasons other than orellanine poisoning, it was found that there was no significant difference between their long-term outcomes, with no additional GI damage or impairment reported in the orellanine group. Taken together, these results support the conclusion that orellanine causes minimal lasting damage to the GIT. This renal selectivity forms a key part of orellanine’s appeal as a potential scaffold for the development of an anticancer agent for use in the treatment of ccRCC.

Nevertheless, the toxicity of orellanine to healthy renal cells remains a significant issue. Here, it causes tubular necrosis, irreversibly damaging the cells of the proximal tubule. This most commonly manifests as pain in the flanks and lower back, along with oliguria.^74^ Even where these symptoms progressed to complete renal failure, biopsy results show that the glomerular barrier remains fully intact.^59,62,75^ The manifestation of such renal symptoms can occur up to 2 weeks after toxin ingestion. Cases with longer latency periods typically display better long-term patient outcomes,^74,76^ although the severity of toxicity is subject to a significant degree of interindividual variation.^71,75^ The fatality rate among strongly evidenced cases in the literature stands at approximately 5.5%, and it is stated that fatalities have seen a further decline in prevalence due to the regular use of hemodialysis as a therapeutic option in confirmed cases of orellanine poisoning.^71^

A combination of the prolonged latency period and general nature of the symptoms, however, has made the diagnosis and confirmation of orellanine poisoning a challenging task. Many patients only present for treatment following the onset of renal symptoms, at which stage mushroom poisoning is no longer an obvious cause.^75,76^ At this stage, orellanine will no longer be detectable in the plasma,^40,66^ with analysis of renal tissue samples currently the only method to confirm a diagnosis of orellanine poisoning.^66^ There is currently no antidote or curative treatment—there have been suggestions that the use of high dose antioxidants with or without steroids may improve outcomes,^77−79^ but such a strategy has failed to produce consistently reproducible results.^80^ Standard therapy focuses on dialysis, to relieve pressure on the kidneys and maximize their chances of recovery.^40,71,81^ Attempts to counteract the oxidative damage with administration of superoxide dismutase (SOD) led to increased toxicity in rat models.^59^ While it was proposed that this occurred as a result of catalase depletion, meaning that the H_2_O_2_ produced by SOD accumulated to toxic levels, this highlights the need for a better understanding of orellanine’s MOA, to allow for rational design of effective treatment strategies in cases of intoxication. A detailed understanding of its MOA could also inform the design of derivatives/targeting strategies that improve selectivity for carcinoma cells over healthy proximal tubular tissue.

## Analysis and Metabolites

### *In Vitro* Snalysis

**Figure 6:**
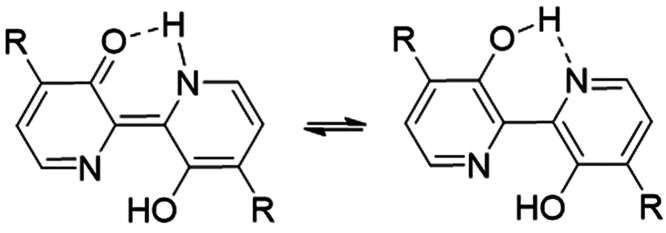
Keto–enol tautomerism of orelline.

As a group of compounds, orellanine and its metabolites have several properties that lend themselves to identification and quantification, by liquid chromatographic methods in particular. While orellanine itself lacks a fluorophore, its reduced metabolite orelline produces a distinctive turquoise fluorescence (excitation wavelength 400 nm, emission wavelength 450 nm)^44,46,82−85^ and is readily formed under a range of conditions, including when exposed to UV light.^40,42−44,46,86,87^ This can be explained by the reduction of the *N*-oxides, which enables the necessary electron spin transitions via keto–enol tautomerism, a phenomenon seen across a range of 3,3′-dihydroxy bipyridines^42,88^ (Figure 6).^88^

Orellanine can also chelate ferrous iron, resulting in the formation of a dark red complex. This has been used in a number of publications to enable sample visualization during thin layer chromatography (TLC) analysis.^46,54,83,85,89^ Both quantitative and qualitative analyses have been reported, with the methods used encompassing TLC,^46,64,66,82,84−86,90^ electron spin resonance (ESR),^85,90^ electrophoresis,^85^ high-performance liquid chromatography (HPLC),^26,41,72,91−93^ gas chromatography-mass spectroscopy (GC-MS),^87,94^ and liquid chromatography-mass spectroscopy/mass spectroscopy (LC-MS/MS).^26,31,65,72,95^ While earlier studies predominantly relied on TLC, HPLC-based methods became the analytical tool of choice following the work of Holmdahl et al. in 1987^92^ (Table 1).

**Table 1:**
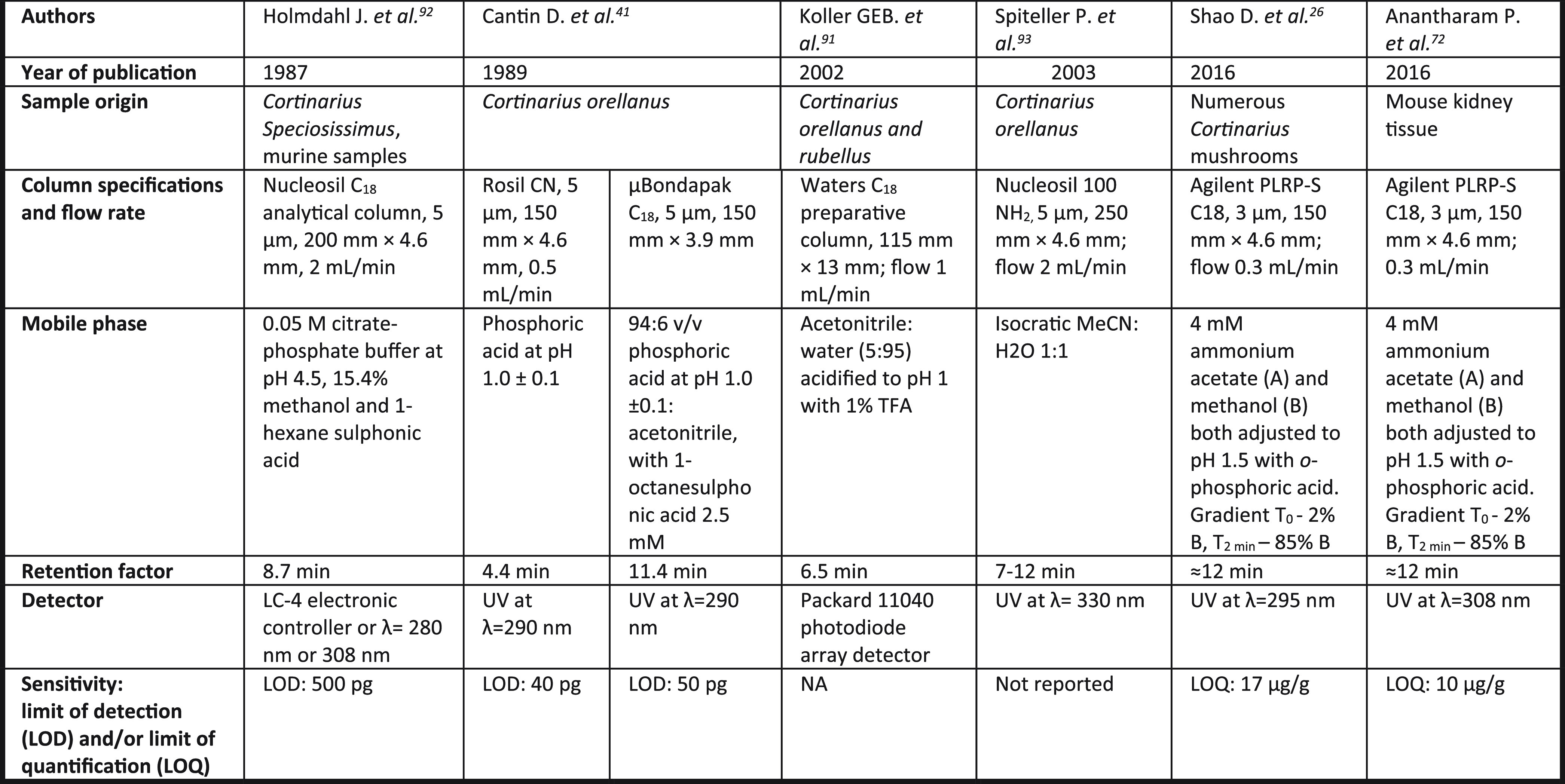
HPLC Methods for the Analysis of Orellanine

These early methods were developed for analysis of orellanine content in mushroom extracts and tended to utilize a C_18_ column^26,41,91−93^ and an acidic mobile phase, preventing ionization of the catechol groups.^26,41,92^ These techniques were largely employed to determine/compare the presence of orellanine in different mushrooms. In 2002, the first LC-MS method for the detection of orellanine was reported^91^ (Table 2).

**Table 2:**
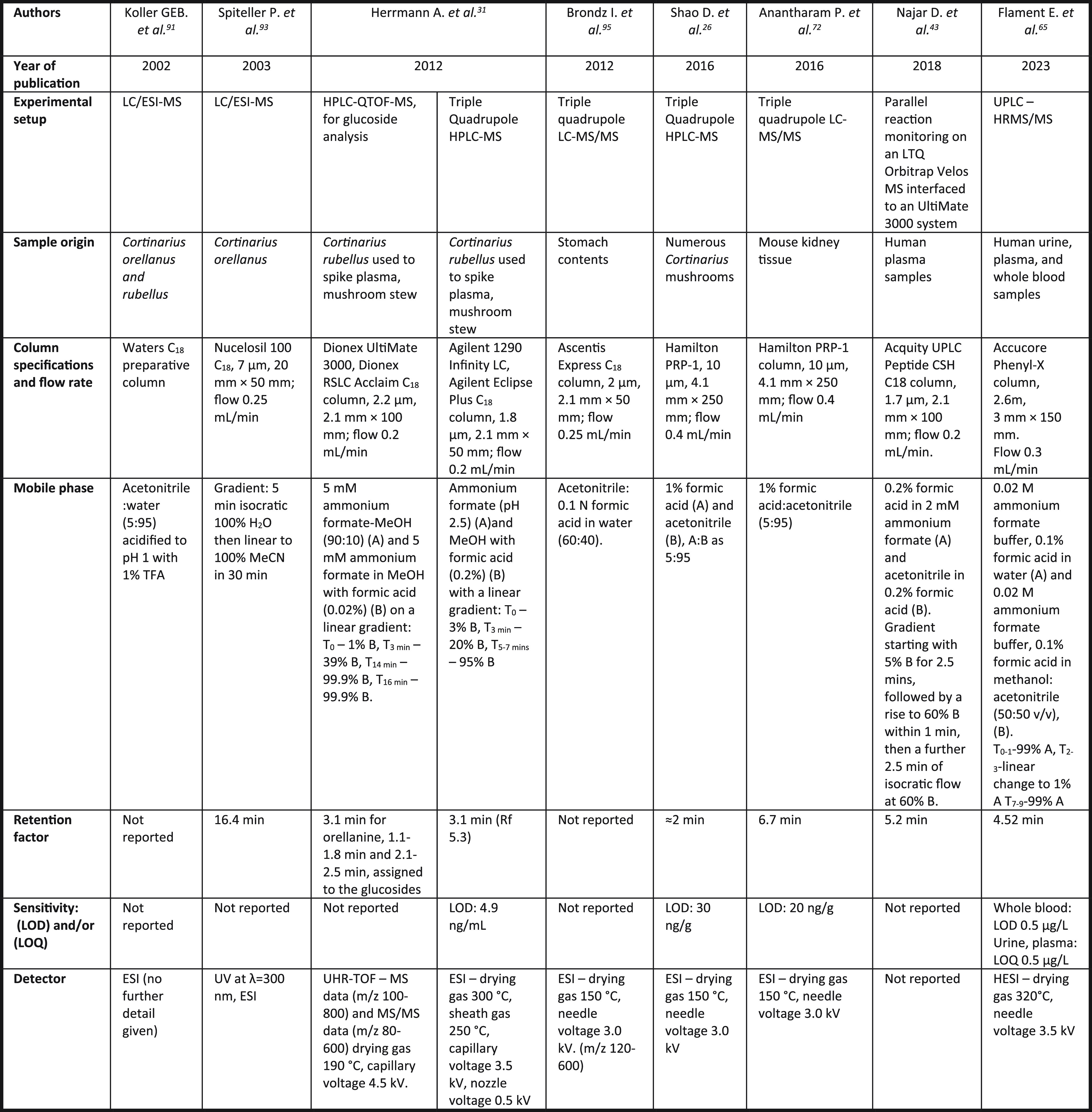
LC-MS and LC-MS/MS Methods for the Analysis of Orellanine

This was followed in 2012 by a method for the analysis of orellanine in human biological matrixes, using LC-MS to detect orellanine in spiked human plasma.^31^ Like the earlier HPLC methods, most of the published LC-MS and LC-MS/MS methods use low pH mobile phases,^26,31,65,72,91^ with the inclusion of a buffer such as ammonium formate becoming increasingly common.^26,31,43,65^ There has also been a move away from the use of standard C_18_ columns in recent methods, with peptide,^43^ phenyl,^65^ and polystyrene divinylbenzene (PRP-1) columns now commonplace.^26,72^ These changes have resulted in techniques that can detect the presence of orellanine in tissue samples at concentrations as low as 20 ng/g.^72^

Despite this, there are a few challenges associated with orellanine analysis. The first is that while analysis of plasma levels is relatively straightforward,^43,64−66,86^ extraction from renal tissue samples is more challenging, with the toxin exhibiting reduced solubility in the intracellular environment.^66,72^ Given orellanine’s rapid plasma clearance and prolonged tissue retention, such tissue sample assays^64,66,72^ have greater applications in a diagnostic setting than the more commonly reported plasma extractions.^31,43,64−66,86^ Likewise, methods to quantitatively assess and compare the uptake of orellanine or orellanine derivatives in proximal tubular cells and ccRCC cells would have value in assessing the selectivity of orellanine-based cancer treatments.

### Role of Metabolites

**Figure 7:**
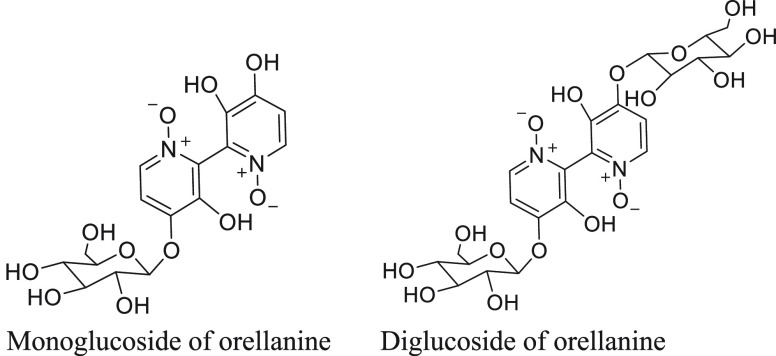
Orellanine mono- and diglucoside metabolites.

Another issue is the solvent conditions used in many of the current methods. Orellanine and its breakdown products are practically insoluble in water and most organic solvents.^37,40,44^ Methanol is one of the few common solvents in which they all show some degree of solubility. This can be further enhanced by use of acidified methanol, doubling the total toxin extraction.^31^ This method was originally used in extraction and quantification of the orellanine content in mushrooms, where a major issue was quickly encountered. In orellanine containing *Cortinarius* mushrooms, the compound exists primarily as the 4,4′-diglucoside metabolite, although both the monoglucoside form and orellanine itself have been detected in samples of *C*. *rubellus*(31,93) (Figure 7),

The acetal bond that links the glucose moiety to orellanine is prone to cleavage under acidic conditions, thus requiring samples to be analyzed immediately after extraction. Another complication is that there is no consensus as to whether derivatization is necessary to enable their MS analysis.^31,93,95^ When orellanine is tested or analyzed in biological samples, this at first appeared not to be a major issue as the acidic solvent conditions used ensured that there was minimal intact glucoside present during analysis. However, during pharmacokinetic studies carried out by Najar et al.,^43^ a cluster of apparent orellanine metabolites were detected via LC-MS analysis in rat plasma samples following orellanine dosing. These metabolites had shorter retention times, eluting after 4 min vs 5.2 min for orellanine. It was also noted that the intensity of the peaks representing these metabolites increased over time, while the orellanine signal diminished. The authors concluded that these metabolites were most likely orellanine glucoside metabolites, formed *in vivo*. They were unable to confirm this hypothesis as the parent ion of these conjugates was not detected during the ionization step. Given their proven role in the metabolism of catechols, it is also possible that the metabolites observed in this study were orellanine glucuronides.^96^

It has long been proposed that at least some degree of orellanine’s action is due to the presence of one or more active metabolites; one of the earlier animal-based studies showed that orellanine was only able to inhibit protein synthesis after it had been exposed to liver microsomes,^97^ while another study found that administration of the metabolic enzyme inducer phenobarbital enhanced the toxicity of orellanine in rats,^98^ suggesting some form of metabolic activation. A more detailed understanding of the nature of these metabolites will be required if orellanine is to be optimized as a potential cancer treatment. Structural modifications or conjugation to a targeting vector can have a significant impact on the metabolism of a molecule—the level of importance that orellanine’s metabolites play in its activity could therefore influence the choice of positioning point for a targeting moiety along its structure.

**Figure 8:**
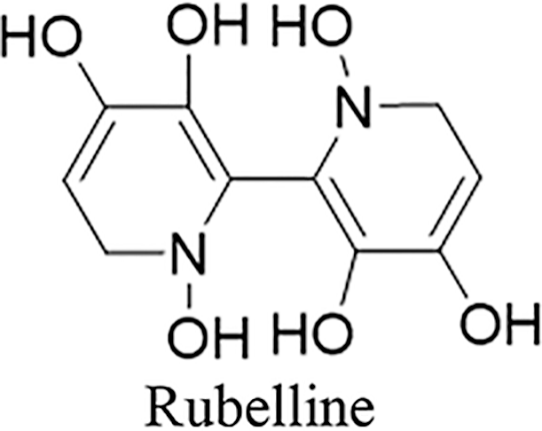
Proposed structure of rubelline.

A counter argument to the proposed importance of the orellanine glucoside was provided by Brondz in 2013.^87^ He stated that his group was unable to detect orellanine glucosides in rat stomach fluid and that this was due to the compound’s instability in aqueous acidic conditions. He concluded that it was unlikely orellanine diglucoside present in mushroom samples could be absorbed intact following passage through the GIT. Instead, he used GC-MS SMB data to propose the existence of a novel toxin, which he dubbed rubelline (Figure 8).

**Figure 9:**
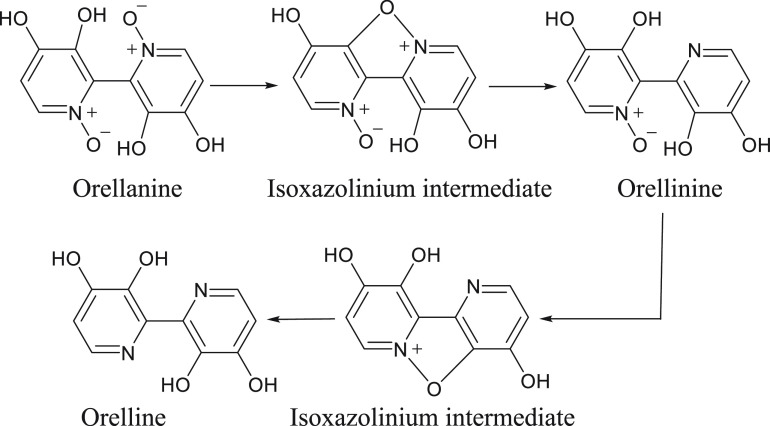
Proposed isoxazolinium intermediates generated during breakdown of orellanine and orellinine.

This was reasoned to be more readily absorbed in the GIT due to its greater degree of lipophilicity. It was further hypothesized that rubelline could be converted to orellanine enzymatically in the kidney, with the rate of conversion depending on the degree of enzymatic activity in each individual. While this hypothesis could in theory explain both the delayed onset and broad range of clinical responses seen in orellanine toxicity, no further data have been reported to support this argument. Another theory states that the toxicity of orellanine is largely attributable to the formation of intermediates during the degradation of orellanine to orelline.^99^ These intermediates are proposed to contain an isoxazolinium core, which could facilitate protein binding, both leading to intracellular toxicity and potentially contributing to the retention of orellanine within the kidneys^100^ (Figure 9).

**Figure 10:**
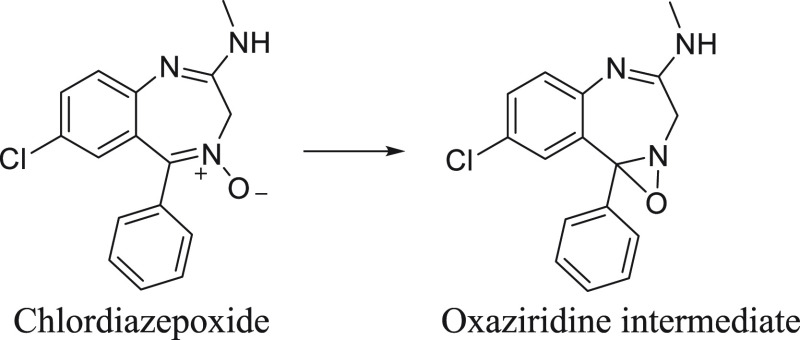
Structures of chlordiazepoxide and its oxaziridine breakdown intermediate.

In support of this hypothesis, orellanine toxicity in an animal model was found to be greater when the compound was reconstituted under light than when it was prepared in the dark.^99^ Although such results are yet to be replicated in any subsequent publications, a similar phenomenon has been observed with the toxicity of orellanine in photosynthesizing plants.^48^ The phototoxicity of chlordiazepoxide provides precedence for this sort of toxic mechanism. Following exposure to UV radiation, the *N*-oxide group in chlordiazepoxide degrades to give an oxaziridine intermediate, which exhibits protein binding and tissue damage, primarily in the skin, liver, and kidneys^100,101^ (Figure 10).

Partially because of this uncertainty as to their exact nature, the isolation of orellanine’s active metabolites has remained elusive. Refinement of the current analytical processes is needed to accurately detect and quantify orellanine and its metabolites *in vivo*.

### *In Vivo* Analysis

Another area for improvement is in the animal models used to assess its MOA and toxicity as many of the present models tend to be rat-based.^43,59,62,67,69,76,98,102,103^ A major drawback of rat models for orellanine toxicity is that rats are known to be more resistant to the toxin than humans.^31,40,67^ Gender-based differences have been reported, with female rats showing greater susceptibility to the toxin than their male counterparts. While females were observed to display damage to the inner cortex as a sole histological symptom, such damage in males was rarer and always accompanied by infiltrates in the outer medulla.^103^ Although it was noted that rats exhibit sex-based differences in enzymatic activity along their inner cortexes, no data was provided to link such variation to the histopathology of orellanine. Some have proposed that murine models are a more accurate mirror to the human response to orellanine.^72^ Mice show several tissue-specific toxicities not seen in rats, including a hepato-renal syndrome that was also observed in some of the older reported cases of orellanine poisoning in humans.^37^ However, it has since been suggested that these symptoms in humans may have been caused by concomitant ingestion of other poisonous mushrooms. Nevertheless, mice also display numerous other symptoms yet to be reported in humans, including damage to the lungs and spleen.^72^ Although no acute toxicity was reported, a pharmacokinetic study of the tissue distribution of tritium-labeled orellanine also found that the liver and spleen displayed the highest levels of orellanine accumulation outside of the bladder and kidneys.^43^ While animal models tend not to map exactly onto the human conditions and systems for which they proxy, the issue in this case is that the physiological underpinnings for these deviations from the human response to orellanine are poorly understood. In order to test and understand the action of orellanine, animal models that can be reliably mapped onto the human response are required.^5^

## Synthesis

**Figure 11:**
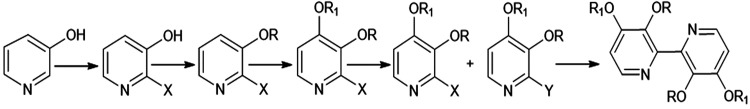
General schematic for the synthesis of tetra-protected orelline X = Cl, Br, or I; R = CH_3_ or CH_2_OCH_3_; R_1_ = CH_3_; Y = X for homocoupling, SnBu_3_ for heterocoupling.

Due to its relative simplicity, synthesis of orellanine can be accomplished in less than 10 steps. The earliest published synthesis of orellanine was by Dehmlow and Schulz in 1985.^104^ Despite resulting in an overall yield of less than 0.5%, the general steps and reagents used in this synthesis were retained by the majority of the subsequently reported orellanine syntheses (Figure 11).

This general method starts with halogenation at position 2 of 3-hydroxy pyridine. While bromide, chloride, and iodide groups have all seen use, the iodide group has become the most prevalently used in more recent methods. This is then followed by protection of the hydroxy moiety. Although the identity of the 4-substituent and the nature of its attachment varies, most methods then proceed to a homocoupling method that was first published by Tiecco et al. in 1986.^42^ This results in the formation of a 3,3′-4,4′ protected bipyridine, which in most methods is tetramethylorelline. Subsequent *O*-demethylation and *N*-oxidation affords orellanine with the order of these steps being flexible. Dehmlow et al. introduced the *N*-oxides as the penultimate reaction, while Tiecco et al. conducted this step last. An overall orellanine yield of 3.9% was reported by Tiecco et al. compared to 0.134% by Dehmlow and Schulz.

The employment of a trimethylsilylethoxymethyl (SEM) protecting group at position 3 of the pyridine by Hasseberg and Gerlach^105^ allowed for progression to the homocoupling step within three steps, as opposed to five and six steps for the methods published by Dehmlow and Schulz and Tiecco et al., respectively, achieving an overall orelline yield of 4.8%. Exploitation of the chelating properties of the SEM protecting group by Hasseberg et al. allowed for differential introduction of substituents at the 4- and 4′-positions after bipyridine formation to generate asymmetrical orelline derivatives. Interestingly, when they attempted to use the method of Tiecco et al. for the *N*-oxidation step, they were unable to synthesize orellanine. Subsequently reported methods only show the synthesis of orelline. This may stem from the fact that this compound has a more desirable stability profile than its oxidized derivative, orellanine, which is photolabile.

**Figure 12:**
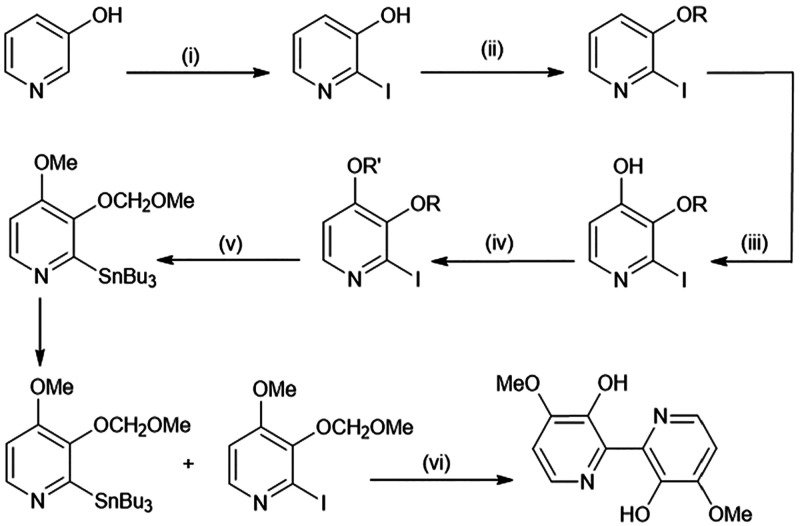
Method for the synthesis of partially protected orelline by Mongin et al.^107^ Reagents and conditions: (i) I_2_, Na_2_CO_3_, H_2_O, 0.5 h, 20 °C, 85%; (ii) R = CH_2_OMe: NaH, MeOCH_2_Cl, DMSO, 15 h, rt, 70%; R = Me: MeONa, MeI, DMF, 15 h, rt, 85%; (iii) 2,2,6,6-tetramethypiperidine, BuLi, B(OCH_3_)_3_, CH_3_CO_3_H, THF, 1 h, −50 °C, then 2 h, −75 °C, 63% (R = Me), 78% (R = CH_2_OMe); (iv) R′ = Me: Ag_2_CO_3_, MeI, THF, 15 h, rt, 71% (R = R′), 69% (R = CH_2_OMe); R′ = CH_2_OMe: Ag_2_CO_3_, MeOCH_2_Cl, THF, 15 h, rt, 71% (R= OMe); (v) BuLi, Bu_3_SnCl, THF, −75 °C for 0.25 h, then rt, 65%; (vi) Pd(PPh_3_)_4_, CuBr, dioxane, 15 h, reflux, acidic workup, 74%.

While poor yields had historically been an issue with orellanine synthesis, progress was made in addressing this problem by Trécourt et al. in 1993 when they devised a synthesis of orelline that used 4-methoxypyridine as the starting material.^106^ They also employed a metalation technique, thus avoiding the use of concentrated acids that were common in earlier methods. This resulted in a significantly improved orelline yield of 15%. One significant drawback of this method was that it lacked the flexibility to allow for the synthesis of asymmetrical bipyridines, thus limiting the potential for future modification or optimization of the orelline skeleton. However, this was addressed by the same group in 2002 with their employment of a different pyridine coupling method.^107^ Instead of conducting a homocoupling reaction, they carried out a cross-coupling reaction, which allowed for pairing of different substituted pyridines to produce a flexible range of 3,3′-4,4′ substituted bipyridine derivatives in reasonable overall yields (∼15%), while also having the added advantage of avoiding the use of diazomethane. At the time of writing, this remains the most efficient method for the synthesis of protected orelline derivatives (Figure 12).

In future studies significant emphasis is likely to be placed on synthetic methods where orellanine can be decorated with protecting groups, which can then be selectively removed to allow for attachment to targeting moieties including antibodies. This is an increasingly popular strategy for selective delivery of small molecules to tumor tissue.^2,108^ However, the harshness of many of the reaction conditions currently utilized in the synthesis of orellanine would pose a major challenge to this approach. Any targeting moieties used would likely be designed to facilitate selective cleavage and subsequent payload release into the environment either around or within the tumor cell. Given the additional storage and handling requirements resulting from the photolability of orellanine, it has typically been preferred to conduct the *N*-oxidation reaction last, regardless of the synthesis pathway. To date, all published methods for the *N*-oxidation of orelline have utilized hydrogen peroxide. It is worth noting that Tiecco et al.^44^ published a method for the synthesis of orellinine from orelline, where they used *m*-chloroperoxybenzoic acid (*m*CPBA) in chloroform to achieve *N*-oxidation on one of the pyridine rings, while suggesting that an excess of *m*CPBA could be used to furnish orellanine. Unfortunately, such conditions could pose a significant challenge where small molecule or protein-based targeting agents have been conjugated to orelline prior to its oxidation. Therefore, the development of a selective *N*-oxidation method that utilizes milder conditions would be an important development for future studies where targeted, orellanine-type conjugates are employed.

Given the nature of the reduction mechanism in the degradation of orellanine,^39^ there is a second approach that could be taken to delay the manifestation of photolability and cytotoxicity until the end of the synthesis. This would entail removing the 3,3′-hydroxy protecting groups after the *N*-oxidation as the last step in the synthesis. While there is precedence for such an approach,^44,104^ the harshness of the conditions required for deprotection under the current methods poses a similar issue with regards to the stability of potential targeting moieties or linker units. The highest reported yields of orelline to date have been when the 3,3′,4′,4′ hydroxies have been protected as methyl ethers. This is in spite of the fact that removal of this protecting group requires the use of elevated temperatures and concentrated HBr. Utilization of alternative 3,3′-hydroxy protecting groups, which could be selectively removed at the end of synthesis under relatively mild conditions, would allow for maximization of the time spent by the product in a more readily workable form.

## Chemotherapeutic Potential

Work to develop refined syntheses and analytical techniques for orellanine has gained an added impetus, with recent studies suggesting that it may have efficacy in the treatment of metastatic ccRCC.^62,63,109^ ccRCC originates from the epithelial cells lining the proximal convoluted tubule^62,110,111^ and accounts for over two-thirds of kidney cancer globally each year.^111^ The 5-year survival rate stands at 12% for distant metastatic cases, with a reported 8.76% of patients presenting with distant metastases upon initial diagnosis.^111^ Current nonsurgical treatment strategies employ combinations of anti-PDL1 checkpoint inhibitors, tyrosine kinase inhibitors, and antiangiogenic agents.^112^ There are currently no predictive biomarkers for disease progression, nor for treatment efficacy in ccRCC. The efficacy of orellanine in such tumors was reported by Hedman^62,63^ and a team of researchers at the University of Gothenburg. As might have been predicted based on its toxicity to proximal tubular cells, orellanine showed antitumor effects in ccRCC cell cultures.^62^ While ED_50_ values for local ccRCC were reported at low micromolar concentrations, there was no major toxicity detected in similarly dosed endothelial (HUVEC), hepatocyte (HEPG2), or breast cancer (MDA) cell lines.^62^ Promisingly, they also reported efficacy in a number of metastatic ccRCC cell lines (i.e., SKRC-17 and SKRC-52), which exhibited increased levels of oxidative markers, reduced mitochondrial function, and increased levels of caspase 8 and 9 mediated apoptosis. This activity was replicated, both in cell lines derived from patient tissue samples and a rat-based xenograft model, where levels of tumor necrosis were nearly 4 times greater in subjects dosed with orellanine.^63^ Several of orellanine’s currently known features underline its potential as a chemotherapeutic agent for patients with ccRCC. Given the selectivity of its action in proximal tubular cells, it has been suggested that there may be a specific transporter responsible for its uptake and retention.^43,63^ This presents an opportunity, in the event of transporter identification, for the establishment of a relationship between the expression of the transporter gene and the levels of orellanine uptake and action. Were such a relationship to be established, this would signify the first biomarker identified in the treatment of ccRCC.^111^ Another advantage of orellanine is that data from toxicology studies have suggested that, as present in nature, it has limited emetic potential^70^ and no major systemic side effects outside of those related to its renal toxicity. Coupled with its novel MOA, this would suggest that orellanine could have potential to be used in combinations with other chemotherapies without significantly amplifying the overall toxicity of the regimen. However, this comes with the sizable caveat that is orellanine’s significant renal toxicity. The recently approved phase I/II trials on the use of orellanine in treatment of metastatic ccRCC^113^ acknowledge this issue by excluding all patients who are not already undergoing dialysis. While cytoreductive nephrectomy and subsequent dialysis are common even in cases diagnosed at the distant metastatic phase,^111^ this nonetheless has the effect of notably reducing the range of cases in which orellanine could be deployed, either alone or as part of a broader regimen. With this in mind, there is clear scope for further development of orellanine as a lead molecule in the search for a novel targeted agent against ccRCC. In this regard, the development of orellanine derivatives capable of discriminating between ccRCC and healthy proximal tubular cells will likely become a priority.

## Conclusions

Before orellanine’s true potential as a therapy can be realized, there are still significant questions to be answered regarding the identification of the specific renal transporters responsible for orellanine uptake from urine into tubular cells, the apparent lack of selectivity for ccRCC over healthy proximal tubular cells, and the precise role, if any, played by its metabolites in its MOA.

While the development of biological agents and improved techniques for the *de novo* design and synthesis of leads have often been seen as removing the need for natural products in drug design, there is another approach that can be taken. Rather than simply replacing natural products, these new techniques can be used to extract value from compounds previously seen as unusable. As exemplified by the success of ADCs, modern drug development techniques can be used to “salvage” natural compounds whose cytotoxicity or pharmacokinetic profiles had previously seen them deemed clinically irrelevant. With the modern drug development process culling so many compounds that produced promising *in vitro* data only to fail to perform in an *in vivo* setting, the benefits of working with a naturally inspired compound that has proven bioactivity in humans should not be underestimated. While orellanine is a particularly promising example, blending of modern drug development techniques with the range of structures and bioactive profiles that natural products provide can ensure that such natural product-based approaches remain a reliable component of the drug development process.
